# Overcoming the phenomenological Perpetuum mobile in clinical cognitive neuroscience for the benefit of replicability in research and the societal view on mental disorders

**DOI:** 10.3389/fnhum.2022.1054714

**Published:** 2022-11-30

**Authors:** Christian Beste

**Affiliations:** ^1^Cognitive Neurophysiology, Department of Child and Adolescent Psychiatry, Faculty of Medicine, TU Dresden, Dresden, Germany; ^2^Faculty of Medicine, University Neuropsychology Center, TU Dresden, Dresden, Germany

**Keywords:** cognitive neuroscience, psychiatry, mental health, replicability, stigmatization, science policy and funding, cognitive science, clinical neurosciences

## Abstract

Cognitive neuroscience comes in many facets, and a particularly large branch of research is conducted in individuals with mental health problems. This article outlines why it is important that cognitive neuroscientists re-shape their role in mental health research and re-define directions of research for the next decades. At present, cognitive neuroscience research in mental health is too firmly rooted in categorial diagnostic definitions of mental health conditions. It is discussed why this hampers a mechanistic understanding of brain functions underlying mental health problems and why this is a problem for replicability in research. A possible solution to these problems is presented. This solution affects the strategy of research questions to be asked, how current trends to increase replicability in research can or cannot be applied in the mental health field and how data are analyzed. Of note, these aspects are not only relevant for the scientific process, but affect the societal view on mental disorders and the position of affected individuals as members of society, as well as the debate on the inclusion of so-called WEIRD and non-WEIRD people in studies. Accordingly, societal and science political aspects of re-defining the role of cognitive neuroscientists in mental health research are elaborated that will be important to shape cognitive neuroscience in mental health for the next decades.

## Introduction

Cognitive neuroscience comes in many facets, and research in this field can roughly be divided into cognitive (neuro)science research that deals with neural principles underlying cognitive functions in “healthy” individuals and individuals characterized by some “disorder” or mental atypicality. With the advent of the Research Domain Criteria (RDoC) framework (Casey et al., 2013; Cuthbert, 2020) at the latest, cognitive neuroscience has gained a central role in research on mental disorders because different aspects of cognitive functioning and their neural underpinnings have been considered helpful for the understanding—or better say the description—of mental disorders. The term “description” should be preferred because an “understanding” requires a more theory-driven, mechanistic approach in the examination of cognitive functions, which is significantly underdeveloped in research on mental disorders and which requires a fundamental change in how cognitive neuroscientists conduct research in mental disorders.

In the following, the “phenomenological Perpetuum mobile” is outlined and which consequences this has for replicability in neuropsychiatric research and the societal view on mental disorders. Attention-deficit-hyperactivity disorder (ADHD) is presented as an example to illustrate the fundamental problem, which, however, also applies to other mental health conditions. Overcoming these problems is one of the future most important tasks for cognitive neuroscientists. Cognitive neuroscientists are in an optimal position to be the driver of these necessary changes beyond cataloging differences between groups of individuals. However, for that to happen, the cognitive neuroscience community should reconsider its role in mental health research.

### The phenomenological Perpetuum mobile–An illustrative example

Research in neuropsychiatric disorders is mainly driven by a phenomenological approach that roots in the necessities of clinical practice. Clinical practice, and diagnostic procedures, in particular, are characterized by an approach in which different symptoms shown by an individual are meticulously collected, weighted, and integrated to derive a diagnosis. While this procedure is necessary for clinical practice, it confers a fundamental problem when basing research questions quite firmly on such diagnostic definitions. This is because the defined phenomenological level and classification of a disorder are subject to considerable changes with consequences for reliable and comparable research over long periods. Take ADHD as an example:

For ADHD, diagnostic criteria have changed considerably in the past, and even though the changes have been deemed to be subtle in some instances, these changes are essential (Epstein and Loren, 2013). What we call ADHD nowadays has been termed Hyperkinetic Reaction of Childhood in the DSM-II in 1968, and excessive motor activity was the main focus of the diagnostic criteria of this disorder. Then, with the DSM-III, considerable changes in the diagnostic criteria were introduced, stressing attentional problems, impulsivity, and hyperactivity, which is why the term Attention Deficit Disorder (ADD) (with and without hyperactivity). What becomes clear here already is that the phenomena of a clinical condition become a different weighting, and additional facets of some generating processes “emerge” or are at least considered relevant. With the advent of the DSM-III-R, “ADHD” was introduced, and ADD without Hyperactivity was eliminated. However, because this was highly controversial, a turnaround was coined in DSM-IV. There, ADHD was retained, and subtypes were introduced (predominantly Inattentive, predominantly Hyperactive-Impulsive, and Combined), providing the option to—again—consider the previously abandoned hyperactivity-impulsivity facet and the facet of inattention in ADHD. This shows how a clinical condition—as defined by phenomena—can “change” considerably in the history of clinical research. However, it did not stop there. With the DSM-5, the introduced ADHD “subtypes” from DSM-IV were reframed as “presentations”, thus weakening the conceptual clarity and is distinctiveness. In addition, other changes occurred. For example, the onset of symptoms was changed from before age 7 to before age 12, and that functional impairments only need to “reduce the quality of social, academic or occupational functioning” instead of requiring that they be “clinically significant.” The briefly sketched process makes perfect sense from a clinical perspective and the necessities related to clinical care.

However, from a research perspective and especially considering the role of cognitive (neuro)science, as stressed in the RDoC framework, the described process makes it hard, if not impossible, to gain mechanistic insights explaining a clinical condition. Opposed to psychiatric research, cognitive neuroscience research is firmly rooted in the tradition of theory-driven research with the impetus to examine mechanisms *behind* phenomena—that is, what generates phenomena. Many breakthroughs in understanding (human) brain function were possible through systematic testing and falsifying theories addressing a particular mechanism behind phenomena. So, mechanistic cognitive neuroscience research starts from the mechanisms behind phenomena, not the phenomena themselves. Mechanistic explanations have to go beyond the widespread custom of relying on “empirical generalization” (Hommel, 2019)–i.e., merely turning clinical observations into descriptions that abstract from the concrete patient and circumstances (Hommel, 2019). Empirical generalizations are necessary in diagnostics, but given their descriptive, non-causal nature, they do not allow identifying the underlying functional and neurobiological mechanisms to the degree that allows understanding of exactly how a clinical condition is generated (e.g., ADHD), why it sometimes produces heterogeneous symptoms, and why it overlaps with other clinical conditions (consider for example the high co-morbidity between ADHD and other disorders). These problems are exaggerated further when the definition and criteria for diagnosing a mental disorder change considerably over time (see the above example). Through re-definitions of diagnostic criteria, the question appears whether cognitive neuroscience findings gained when let us say, the phenomenological definition “A” governing the diagnostic procedure can still be held when the phenomenological definition “B” of the diagnostic criteria was in effect. Below, three inter-related topics conferred by the dynamics exemplified above are detailed, which I call a “phenomenological Perpetuum mobile”. The metaphorical implication of this term is clear: Is knowledge and insights gained still valid when another phenomenological (diagnostic) definition is in place, or does another definition automatically necessitate repeating all that has been done before because it may not hold given the new definition of a clinical condition? The problem is that this question is unlikely to be answered through only “re-doing” empirical research. Therefore, it is important to re-think the cognitive neuroscience approach in the mental health field. Overcoming the outlined issues will require a more robust mechanistic theory-driven approach, requiring cognitive neuroscientists to take a more active role in shaping research on psychiatric disorders.

### Topic 1: Replicability and comparability of findings

Basing cognitive neuroscience research on changing diagnostic criteria and defining categories between healthy vs. ill individuals/conditions based on the outcome of the diagnostic process has clear consequences for the replicability of findings. Not least because of the “replication crisis” (Zwaan et al., 2018), this aspect deserves special attention. Mostly, the causes of the replication crisis have been referred to as underpowered studies, publication bias, problems with applying statistical procedures, and imprecise theories (Lewandowsky and Oberauer, 2020). What has not been that prominently considered—but is of utmost importance in clinical cognitive neuroscience—is the possible shaky ground rooted in changes in the definition of mental disorders. These shaky grounds, however, become pretty apparent when considering the historical development of diagnostic criteria as exemplified in the ADHD diagnosis (see above). However, even worse, the diagnostic process to derive a diagnosis (e.g., ADHD) is also not as consistent as it should be—not only when taking the perspective on the requirements for reliable cognitive neuroscience research. Just recently, it has been shown that there are considerable variations in both the symptoms and the diagnosis of ADHD even within a country (Widding-Havneraas et al., 2022). It has also been shown that clinical data from medical health records perform only poorly in machine learning approaches to classify ADHD individuals (Mikolas et al., 2022), which contrasts with findings using more theory-driven experiments and cognitive neuroscience data (Vahid et al., 2019). All this adds to known problems that even though mental disorders can be considered global phenomena, they are subject to strong modulatory effects related to historical, cultural and political factors impacting diagnostic procedures (Smith, 2017). This is not only the case in the chosen example but applies to many other psychiatric conditions (Canino and Alegría, 2008; Alarcón, 2009). All these aspects underline a “random” or at least not-well controllable component to diagnosing mental disorders. So, what measures can be taken to provide more solid grounds for cognitive neuroscience research in mental health conditions?

One means proposed to counteract replicability problems lies in the “#Manylabs” efforts in various psychological science and neuroscience fields (Open Science Collaboration, 2015; Klein et al., 2018; Pavlov et al., 2021). The term “#Manylabs” refers to initiatives in which previously published experiments/studies are replicated through the coordinated effort of different labs. That is, many laboratories are working together and replicate a previous study and data collection is performed in different labs with a clear *a priori* consented study protocol that reflects the methodological approach from the original (to be replicated) study. While this is, in principle, a practical approach, the considerable variations that are even evident within a country jeopardize such efforts from the beginning on because the “#Manylabs” rationale requires that there are minimal variations between the sites/laboratories in the conduction of tests and the inclusion criteria of individuals into a study. As briefly outlined above, consistent inclusion criteria seem to be hard, if not impossible, to guarantee, and many uncontrollable factors can have an impact. Therefore, it is likely that a “#Manylabs” strategy, if based on the phenomenological diagnostic criteria, cannot solve the problem.

Another popular means to counteract problems of replicability has been suggested to be conferred through the pre-registration of studies and registered reports. Compared to the “#Manylabs” strategy, a pre-registration and a registered report can at least partly circumvent the problem imposed by regional and country differences in diagnosing mental disorders and the use of the diagnosis as a starting point for cognitive neuroscience research because the pre-registered study can be conducted at only one study site. However, recent analyses have shown that this strategy is not without problems (Ulrich and Miller, 2020; Miller and Ulrich, 2022), and these problems are particularly central when it comes to cognitive neuroscience research in mental health:

A central problem that pre-registrations and registered reports cannot overcome refers to the “base rate” of phenomena. The base rate reflects the probability that a sought-after effect is truly present in the population (Miller and Ulrich, 2022). The base rate is not an aspect of a specific study but of the entire research area in which the study is conducted and has a tremendous effect on the probability of obtaining a (non-replicable) false positive result (Miller and Ulrich, 2022). Notably, the base rate is high in fields with a solid theoretical and empirical background. This is because a solid theoretical background allows one to put forward hypotheses about which effects are to be expected and how these effects can be measured (Smaldino and McElreath, 2016; Wilson and Wixted, 2018). When there is a high base rate (e.g., in the field of aging research), there is a high chance of obtaining positive results. Opposed to clinical cognitive neuroscience research in mental health, research on aging is less dependent on changing diagnostic definitions (cf. example in ADHD). Generally, the base rate is low in areas in which studies test for effects that are not evident—probably because of ill-defined aspects in the phenomenon to explain. This is also the case for areas with a less stringent or developed theoretical background (Smaldino and McElreath, 2016; Wilson and Wixted, 2018), because there, researchers are forced to put forward hypotheses based on intuitive or anecdotal knowledge (Miller and Ulrich, 2022)—as derived from phenomena abundant in clinical neuroscience. In this regard, and also referring to the introductory ADHD example, it is interesting that a thorough analysis of existing psychological theories has shown that current psychological theories of ADHD are immune to empirical testing and thus irrefutable (Johnson et al., 2009). It has been argued that the base rates in many areas of psychology can only be estimated, but maybe 20% or even less (Miller and Ulrich, 2022). As conclusively shown by Miller and Ulrich (2022), the base rate strongly affects the rate of false positive findings, with these becoming less likely if the base rate is high. Miller and Ulrich (2022) showed that with typical values of the alpha level and power in cognitive neuroscience or psychological studies, more than half of statistically significant findings would be false positive if the base rate is lower or equals 10%. It is easy to see that shaky grounds related to variations in the diagnosis of mental health conditions are unlikely to provide reasonable grounds to assume that cognitive neuroscience research in the context of mental health conditions operates in a field with high base rates. Therefore, a central task for cognitive neuroscience research in the mental health field is to find more suitable (less shaky grounds) to conduct research.

The discussion about the base rate’s impact on the probability of obtaining a false positive finding provides a possible strategy for getting there. As mentioned, the base rate is higher when a more solid theoretical background motivates studies. This suggests that cognitive neuroscientists should revert their current strategy and start cognitive neuroscience research in the mental health field not based on or strongly oriented at clinical phenomena. Instead, they should use overarching and well-established/elaborated theoretical frameworks for their research. The critical point is that when using a theoretical framework from basic cognitive (neuro)science, i.e., a framework that has been established thorough basic (non-clinical) cognitive neuroscience, one has a useful starting point to transfer this framework to questions of clinical cognitive neuroscience. The essential point is that an already validated and well-corroborated framework from basic cognitive science is transferred into a clinical setting to explain clinical phenomena. Such a validated framework confers a high base rate and thus reflects a suitable starting point. This is at the core of the so-called “synthetic approach” which is detailed as part of the next topic and which is promising for clinical cognitive neuroscience.

The above discussion was centered around direct replications of cognitive neuroscience findings in the mental health field. From a scientific-theoretical perspective, direct replications (i.e., repeating experiments in the same way and population as they were initially conducted) have to be distinguished from conceptual replications (Schmidt, 2009). However, the latter is not prominently discussed and rarely conducted because they are deemed riskier and more effortful (Schmidt, 2009). Conceptual replication means a previous research idea/finding/hypothesis is tested using different methods. This requires that a solid theoretical framework is placed and transferred to questions relevant to cognitive neuroscience research in mental health. Importantly, it is then also necessary to think about how cognitive neuroscience methods can best be connected to the research question asked and the theoretical framework used, as has recently been discussed elsewhere (Beste, 2021).

### Topic 2: De-stigmatization of mental health conditions

The above discussion already shows that changes in how cognitive neuroscience research in mental health is conducted are necessary and that a possible change should include a stronger theory-based starting point of cognitive neuroscience research in mental health. This implies essentially reverting the starting point of research efforts from phenomena to theory and overarching concepts. Importantly, this is not only beneficial for aspects including the replicability of findings in the field. It is also of fundamental importance when being interested in sustainably reducing stigmatization associated with mental health conditions. Changes in the research practice of cognitive neuroscientists can contribute to the de-stigmatization of individuals affected by mental health problems. It will become clear that because cognitive neuroscience can be stronger theory-driven in the mental health field, cognitive neuroscientists can play an active role in practical efforts to reduce the stigmatization of individuals affected by mental health problems. A change in the research strategy outlined in Topic 1 will also have considerable consequences on how society looks at individuals with mental health problems and how these affected individuals can be helped to find their place in society.

According to Corrigan et al. (2014), different types of stigma can be distinguished: The “public stigma” refers to negative attitudes other than the affected individual has about mental illness. The “self-stigma” refers to the negative attitudes people with mental illness have about their condition. The last form, the “institutional stigma”, refers to problematic policies of governmental organizations and intentionally or unintentionally limited opportunities for people with mental illness. Thornicroft et al. (2016) summarized knowledge about measures that can effectively reduce stigma in mental health. They show that attitude changes and the improvement of knowledge at the population level (cf. public stigma) have positive effects. It is precisely the knowledge improvement where cognitive neuroscience, if changed to more theory-driven research that can contribute to reducing public stigma in mental health. A stronger theory-based starting point of cognitive neuroscience research confers the benefit that the processes and variations thereof between individuals become the focus of research. The important point to focus on is that people suffering from mental health problems do not show “different” processes. They just show a variation of the same processes that also govern “normal” cognitive function. Through more theory-based cognitive neuroscience research, categorial boundaries between “ill” and “normal” become increasingly fleeting and less distinguishable. As opposed to the current strategy to first categorize individuals into normal and abnormal based on diagnostic procedures that are subject to considerable variation (see above), a so-called “synthetic approach” (Hommel and Beste, 2021; Colzato et al., 2022a,b) for cognitive neuroscience research may be more promising. This approach can be contrasted with the so-called “analytical approach” (Braitenberg, 1986; Hommel and Colzato, 2015). The latter one is the most commonly used in cognitive (neuro)science and psychology (Eronen and Bringmann, 2021) and reflects the strategy to translate real-life phenomena (incl. what is shown in disorders such as ADHD) into a scientific definition, which is later split into definitions of subfunctions (e.g., attention, memory, action etc.) and mapped on underlying processes. The synthetic approach asks, which aspects of different phenomena (e.g., shared aspects between ADHD and other categories of disorders such as autism spectrum disorder, obsessive compulsive disorder etc.) are accounted for by specific theory-defined processes with the goal to account for many phenomena by a the same (limited) set of processes (Hommel and Colzato, 2015). A “synthetic approach” implies that basic processes are likely to contribute to multiple subfunctions and phenomena (Braitenberg, 1986; Hommel and Colzato, 2015). Think about the diagnostic definitions for ADHD referring to “inattention” (e.g., difficulties with “staying focused on tasks or activities”, “starting tasks but quickly losing focus”, “avoiding tasks requiring sustained mental effort”) and symptoms of “*hyperactivity/impulsivity*” (e.g., “having problems in prospectively organizing timelines”, “being always “on the go” as if driven by a motor”, “blurting out an answer before a question has been finished”, “often interrupts or intrudes on others”, “having difficulties waiting for his/her turn”). The synthetic approach asks about a common mechanism behind many of these problems related to “inattention” and “hyperactivity/impulsivity”. Opposed to this, the more analytical approach (as currently evident in the RDoC framework) asks questions about each symptom without trying to find overarching principles that may underly several symptoms. According to the synthetic approach, research begins with one or more specific, well-understood mechanisms combined to reconstruct the phenomena one aims to explain. This will not capture the entire phenomenological spectrum of a mental health condition but only parts of it. It is precisely this dimensional impetus that overlaps between currently existing categorical definitions of mental health conditions stressed in contemporary psychiatric research (cf. RDoC). However, an essential advantage of a synthetic approach is that shaky diagnostic categories no longer hamper cognitive neuroscience research in the mental health field, because it starts from a well-validated cognitive theory that has been built using basic cognitive (neuro)science research and is then transferred to a clinical setting. Using an approach based on theoretically well-defined cognitive neuroscience mechanisms to reconstruct mental health phenomena would be more helpful and generate less and easier-to-understand heterogeneity. Especially the latter aspect is of considerable importance when trying to reduce stigmatization because it is a change in attitudes and knowledge that can effectively reduce the stigma of mental health conditions (Thornicroft et al., 2016). A crucial aspect to consider in the strategic shift and the reversal of cognitive neuroscientists’ research strategy in the mental health field is probably toward a more synthetic approach contributing to a de-stigmatization of individuals with mental health problems in the analysis of the data. At present, data analysis strategies almost wholly follow the traditional approach of classical significance testing with the “goal” of statistically pinpointing *differences* between groups of individuals. When considering stigmatization, the emphasis on differences between groups counteracts efforts to reduce public stigma. How can public stigma be reduced if researchers focus on differences between groups of individuals? Logically, this is impossible, especially when considering the oftentimes inadequate equation between significance and relevance or meaningfulness. Sometimes, empirical research follows the simplistic statement “Significant is significant, regardless of size”. This view has to be qualified and, indeed, there are many criticisms against mere significance (null hypothesis) testing without considering effect sizes. Common misconceptions, even amongst scientists when it comes to differences between groups of individuals in null-hypothesis testing. Three major misconceptions of the *p*-value refer to the classification, replication and magnitude fallacies (Hentschke and Stüttgen, 2011):

1. Classification fallacy: Opposed to this fallacy, a *p*-value does not account for the evidence for a null hypothesis, but rather against it. Therefore, the absence of evidence is not evidence of an absence of effects (i.e., differences between groups of individuals).
2. Replication fallacy: Opposed to this fallacy, the *p*-value does not indicate the probability of replication in the sense that a smaller *p*-values indicates a higher probability of replication. This is because the *p*-value depends on the effect size and sample size. These aspects are also problematic when it comes to the aspect of de-stigmatization. In large samples tiny differences become significant and should not be treated and convey as reflecting substantial (i.e., meaningful) group effects.
3. Magnitude fallacy: Opposed to this fallacy, the *p*-value itself says little, because it is determined by the type of testing (parametric vs. non-parametric), on the design of the study (dependent vs. independent study designs), plus aspects related to sample size and effect size (see above).

All these aspects are important when it comes to the question of whether there are meaningful differences between groups of individuals suffering from mental health problems. An oversimplified public interpretation of differences between groups of individuals, likely originating from the above fallacies committed by scientists, is directly counteracting efforts to de-stigmatize individuals with mental health problems. It is the responsibility of researchers and publishers to engage in a more nuanced presentation of research findings better considering the meaningfulness of obtained “significant differences” and how this may be perceived in the public. Having a very strong theoretical prediction and finding no effect makes the whole thing more meaningful—probably more than having a nominally significant finding in a study that is based on merely anecdotal phenomena abundant in clinical neuroscience. Hence, the difference it in importance between finding an effect or not is moderated by the presence or absence of strong theoretical predictions and can be then very useful to reduce stigma.

Current research practices and data analysis are often incompatible with reducing the stigma of individuals with mental health problems. Stressing differences between groups (especially if they are very subtle and only formally “significant”) in research makes it difficult, if not impossible, to credibly work toward a de-stigmatization of individuals affected by mental health problems. Suppose cognitive neuroscientists want to contribute to efforts aiming at a de-stigmatization of an individual with mental health problems—which they should. In that case, it is not only necessary to stress theorizing more, but it is also essential to re-think and re-organize data analysis strategies. Aside a more nuanced presentation of findings going beyond the significant vs. non-significant dichotomy, two possible avenues should be stressed:

A first approach is to actively counteract publication bias and the “file drawer problem” and invest more efforts to publish so-called “negative findings” (i.e., findings where no significant difference between groups has been obtained). The term “negative finding” is undoubtedly a problem because it often, but unjustifiably has the flavor of a “2^nd^ class finding” or less impactful finding in the scientific community. However, this is not the case if the study is well-powered and the appropriate statistical methods have been used to make a clear point (e.g., Bayesian data analysis). Taking the perspective of individuals with mental health problems would represent a big step forward if the cognitive neuroscientists working in mental health would more actively pursue publishing findings of “negative” and “null” results. This will put the cognitive neuroscientific community in an active position to increase their societal impact to benefit patients suffering from mental health problems. Currently, scientific publishing is biased toward presenting mind-blowing differences between groups of individuals. Even though scientific publishers are often stressing their responsibility and ethics in publishing for the benefit of affected individuals, there is still too little room to publish such “negative findings” (i.e., no significant group differences). One possible reason relates to the scientific “culture” of publishing and citing research. It has been shown that there is a strong bias to cite positive results (i.e., results showing a “significant” difference between groups) (Duyx et al., 2017). At this place, please remember the problems associated with the dichotomy of significant vs. non-significant findings briefly discussed above. As long as citations are the most important metric to assess the “quality” or “impact” of a journal, the citation culture of “negative” and “positive findings” confers a problem. However, also high-impact factor journals (e.g., at Nature publishing group) acknowledge these problems by signing the “DORA statement”. Any research result is “positive” if the study has been conducted properly and has a precise theoretical framing. It is a matter of whether finding a non-significant difference between groups of individuals is “negative”. It is very positive because it may be helpful to counteract the stigmatization of individuals with mental health problems.

A second, probably more radical approach, but partly related to the aspects mentioned above, is changing current data analysis strategies. This has recently been proposed by Ince et al. (2022). The central point Ince et al. (2022) make also refers to the problems regarding the replicability of findings, that is, the existence of an effect (see discussion on the base rate as well as the criticism on classical null hypothesis testing). Ince et al. (2022) argue for the calculation of the population prevalence (i.e., the proportion of the population that would show the effect if tested in this experiment) as an alternative approach to analysing data. In this approach, each individual is tested not only once but serves as its own control in repeated testings. While this may not seem much different from single-case studies, the critical point is that through the usage of, e.g., bayesian statistical methods, a generalization of within-participant results to the population prevalence is possible (Ince et al., 2022). For the aspect discussed in the present article, this is of particular relevance because cognitive neuroscience research in conditions where only a few patients can be examined becomes well-feasible and reliable. More important, however, is that when calculating the Bayesian population, prevalence data are not pressed into a binary result (significant or not) (Ince et al., 2022). Using this approach, it is thus possible to better delineate the diverse spectrum or shadings of cognitive neuroscience processes in individuals affected by mental health problems. Oftentimes, significant differences between groups, as obtained in common data analysis procedures, are small. Nevertheless, they are interpreted and, even more important, *perceived* by the public as an indicator of differences that can give rise to dynamics ultimately leading to stigmatisation of individuals with mental health problems. Re-thinking strategies and procedures how to analyze data is therefore needed.

### Topic 3: The role of WEIRD and non-WEIRD people

The above discussion already pointed to differences between groups as a focus on cognitive neuroscience research in mental health, and it became clear that current phenomenological definitions as a basis of cognitive neuroscience research are problematic. An aspect related to these topics is the heavy reliance of studies on so-called “WEIRD” people, i.e., people coming from western, educated, industrialized, rich, and democratic societies. Problems related to this topic in behavioral sciences and related disciplines have been known for a longer time (Henrich et al., 2010) and have important consequence for generalizability of findings and the mechanistic insights obtained. However, this has still not been sufficiently considered in cognitive neuroscience in general and in the context of clinical populations in more particular. There is a long-established debate on the role of cultural factors in psychiatric diagnosis (Alarcón, 2009). Again, think about the ADHD example. Depending on the culture and societal background the same degree of problems related to “inattention” (e.g., difficulties with “staying focused on tasks or activities”, “starting tasks but quickly losing focus”, “avoiding tasks requiring sustained mental effort”) and symptoms of “*hyperactivity/impulsivity*” (e.g., “having problems in prospectively organizing timelines”, “being always ‘on the go’ as if driven by a motor”, “blurting out an answer before a question has been finished”, “often interrupts or intrudes on others”, “having difficulties waiting for his/her turn”) can be considered to be more or less inappropriate. If a psychiatric diagnosis is over-formed by cultural aspects, the more theory-driven synthetic approach is also relevant. As discussed in Topic 2, the theory-driven synthetic approach starts at the level of well-understood mechanisms behind phenomena such as the symptoms of ADHD listed above. Suppose the phenomenon is subject to cultural modulations. In that case, the synthetic approach is also useful for overcoming a bias in the field that has emerged through the predominant inclusion of WEIRD compared to non-WEIRD people in cognitive neuroscience studies. A theoretical framing of processes is unlikely to be prone to a systematic basis conferred by the studied population. However, one may argue that theoretical frameworks which can be used in a synthetic research approach have been built on data from experiments with “convenience samples” (Henrich et al., 2010) and thus carry an inherent bias that is because basic cognitive science research heavily draws on undergraduate samples which are not representative. Therefore, this may pose a problem when using theories built on such studies. However, this problem may be less relevant than it seems to be because there is no unidirectional relation between data and theory (Morrow, 2014). Instead, a theory also affects how data is collected and analyzed: (i) concepts inform the data collection process by determining what data is collected to best capture the theoretical content. (ii) They direct research by hypotheses (based only on the theoretical concept and not necessarily on person-related factors). (iii) They help constrain the conclusions drawn from empirical research (Morrow, 2014). It has been discussed whether the inclusion of a “cultural axis” or dimension may be meant to counteract the problems related to the inclusion of specific samples in research. However, it soon became apparent that it is not appropriate and feasible to include all possible relevant facets of the societal/cultural impact on a single dimension. Moreover, it is argued that it is impossible to “dimensionalize” culture (Alarcón, 2009). To cope for these problems in a practical way, one solution may lie in the reliance on the above-mentioned synthetic approach, combined with a “#Manylabs” strategy: Taking a validated theoretical framework at the basis, different labs in the world could work together. Since it is the conceptual framing and not a phenomenon that is put into the focus, problems related to minimal variations in the phenomenological diagnostic criteria between sites become less of a problem. Instead, variations between the sites become essential to shape mechanism-based cognitive neuroscience research in mental health with the goal to understand facets of individuality.

### A possible solution lies in the reliance on cognitive theory

From the above, it becomes clear that the current way cognitive neuroscience research is practiced in psychiatry is not very helpful from many perspectives. Chances are high that research has to be repeated over and over again—as frequently as diagnostic definitions of mental disorders change. Importantly, even if this was possible (e.g., terms of funding opportunities etc.), chances are high that replicable findings (or the conduction of replication studies) are challenging to obtain. Moreover, the socially relevant aspect of stigmatization and the need for a de-stigmatization of mental disorders may be hampered.

As mentioned, a possible solution to both problems would be to reverse the way of thinking and cognitive neuroscience research practice. While the RDoC initiative has motivated new thinking into dimensions of mental atypicality, one problem in this framework is that RDoC has defined five “domains” supposed to reflect a brain showing dysfunctions to different degrees in various mental health conditions (Casey et al., 2013). The problem with this approach is that due to their complexity, neural processes are often only weakly conceptually constraining (Niv, 2021). Therefore, cognitive neuroscience research has to go even further. Reverting the strategy of cognitive neuroscience research would mean not starting at the phenomenological level (or diagnostic schemes) but the level of some mechanisms specified in well-testable and behaviorally validated theories. An approach is needed in which theoretical frameworks are put into the center of cognitive neuroscience research and organized research efforts. Such theoretical frameworks must then consist of a small set of operating principles that are (a) defined in a coherent, refutable theory; (b) based on functional neuroanatomical and neurobiological systems relevant to a mental disorder; (c) reliably measurable to allow the quantitative assessment of the underlying mechanisms. The mentioned synthetic approach may be a means to do so. Before an example of how this can be accomplished in neuropsychiatric disorders is provided, it is outlined how far such an approach in cognitive neuroscience research will have considerable and touch scientific understanding in the field as well as clinical practice:

1) The existing phenomenological definition/classification of mental disorders makes it hard to integrate the considerable heterogeneity of symptoms and their fluctuations and to understand and predict the relationship and boundaries between one particular condition and other psychiatric conditions. Moreover, questions of replicability are evident. A mechanistic psychological theory allows one to overcome these problems: individuals can then be grouped and targeted concerning basic processes that are dysfunctional rather than observable symptoms that may or may not indicate a shared problem. Aside from aspects contributing to a better de-stigmatization of individuals affected by mental health problems, such an approach may also impact problems of replicability outlined above and the problem of the “base rate” in such efforts. As outlined, the base rate is not an aspect of a particular study but a research field (Miller and Ulrich, 2022). With an approach based on previously validated cognitive science theories as a starting point, the base rates to be considered when planning studies are better known because these refer to the validated theory explaining a phenomenon. Moreover, having a very strong theoretical prediction and finding no effect makes the whole thing more meaningful. Hence, the difference it in importance between finding an effect or not is moderated by the presence or absence of strong theoretical predictions and can be then very useful to reduce stigma.
2) Replacing the existing phenomenological definition/classification with a mechanistic approach will also likely change the analytical logic of viewing psychiatric dysfunction. As mentioned, the phenomenological approach begins with categories based on observable behavior and anamnestic reports that may or may not be distinct and may or may not capture behavioral symptoms related to the same cause. The danger of this approach lies in its top-down nature: the probability of detecting any coherent underlying mechanism hinges on the validity of its categorization–which may often not be given due to its commonly a-theoretical nature. A mechanistic approach comes with a robust bottom-up flavor that does not respect or require any categorization: it specifies the optimal functioning of a process. It determines the individual deviation from the “optimum” –irrespective of the suspected psychiatric conditions of a patient.
3) Focusing on dysfunctional processes rather than observable symptoms will allow the field to replace the categorical view on individuals (“healthy” vs. “ill”, disorder A vs. B) with the often demanded, more dimensional view, according to which one or multiple basic processes that are also in place in so-called “healthy” populations. From that perspective, “ill” or “disorder” categorizations are no longer needed and will pave the way for genuinely dimensional psychiatry. One might ask that the stressing of a mechanistic approach is required given the Research Domain Criteria (RDoC) project. First, the RDoC-developers emphasize that the actual RDoC matrix is tentative and “that these particular domains and constructs are simply starting points that are not definitive or set in concrete.” Notably, while dimensional, the actual RDoC matrix is still relatively less mechanism-oriented than a synthetic approach. Second, while RDoC begins with current understandings of behavior-brain relationships and links them to clinical conditions, it is a much broader data-driven approach without a precise focus on fundamental mechanisms and observable clinical symptoms. Third, the aspect that mental health conditions, if conceptualized as variations from basic overarching mechanisms underlying various instances of cognitive function, individuals can then be described regarding basic processes shared by healthy individuals. This can provide a better basis to change public attitudes, effectively reducing the stigma of mental health conditions (Thornicroft et al., 2016).
4) A focus on overarching theoretical concepts may also be helpful considering the issue of the representation of WEIRD and non-WEIRD people in studies. Opposed to a diagnosis, based on specific phenomena, a theoretical framing of processes is unlikely to be prone to a systematic basis conferred by the studied population. Since it is the conceptual framing and not a phenomenon that is put into the focus, problems of “Manylabs” approaches related to minimal variations in the phenomenological diagnostic criteria between sites become less of a problem. Instead, variations between the sites become essential to shape mechanism-based cognitive neuroscience research in mental health with the goal to understand facets of individuality.
5) In contrast to a phenomenological definition/classification, which rarely provides theory-based guidance to identify suitable treatment, a mechanistic approach has direct implications for education, training and treatment methods, promoting transparent, well-motivated evidence-based treatment programs. The successful demonstration of a link between a mental disorder and a dysfunctional mechanism, as stated by a cognitive (neuro)scientific theory, would open various possibilities for the development of treatments.
6) A mechanistic theory-driven approach will also set the stage for developing more objective and mechanistically sensitive diagnostic procedures. More specifically, the diagnostic procedures, such as being developed directly from the theoretical framework, assess the problem underlying a mental disorder rather than an observable symptom and provide data that can be formalized in mathematical models. This will set the stage for future studies in computational psychiatry and model-based imaging, which will further increase our understanding of the neural bases of a mental health condition. Together with those mentioned above mechanism-based interventional approaches, this would reflect a complete mechanistic translation spanning diagnostics and therapy. The direct relation between diagnostics and therapy will reflect a fully integrated treatment approach to mental disorders.
7) Finally, moving from a categorical to a more mechanism-based dimensional approach is an essential precondition for better understanding individual differences in mental disorders. Concerning patients, this will allow genuinely personalized medicine and treatments that are fully informed by the nature of the individual’s dysfunction. This will optimize the respective processes for healthy individuals, even those who do not pass thresholds of categorical psychiatric conditions. This will enable individualizing both patient treatment and responsible cognitive enhancement in non-patients.

### What makes a good mechanistic theory for clinical cognitive neuroscience research?

In this section, I briefly outline criteria for a good theory because this is central when considering of how to decide for a suitable theoretical framework when doing cognitive neuroscience in the field of mental health. Generally, several aspects of theory should be considered (Gieseler et al., 2019) (see Table 1):

**TABLE 1:** Definitions of a good theory when being brought to cognitive neuroscience research in mental health (modified from Gieseler et al., 2019).

Criterion	Definition
Consistency	Relevance to various observations.
Precision	Clearly defined concepts and operationalization with little room for “stretching”.
Parsimony	There a few mechanistic principles put forward in the theory.
Generality	Favor high explanatory breadth and observations obtained from laboratory experiments and/or real-world settings (incl. clinics).
Falsifiability	Assumptions can be formulated making it possible to make observations prohibited by theory.

Especially the “precision” and “parsimony” aspects require further elaboration. As discussed previously (Beste, 2021), cognitive neuroscience research should particularly consider the “precision” aspect, because research methods in cognitive neuroscience address different levels of inspection—from the single cell to circuitry to systems. In addition, the methods will vary with respect to temporal and spatial resolution (e.g., electrophysiological or BOLD-signal related methods). Cognitive science theories, which are mostly based on behavioral data, vary in their specificity and breadth of neural processes upon which these theories can be applied. It is thus central to critically evaluate whether a specific conceptual (theoretical) framing is well-suited for the method(s) being used in clinical cognitive neuroscience. Considering the “parsimony” aspect, it has recently been argued (Hommel, 2019) that it is central to acknowledge that a theory has to describe how a phenomenon emerges from the interplay a basic elements: *“A mechanism is a structure performing a function in virtue of its component parts, component operations, and their organization. The orchestrated functioning of the mechanism is responsible for one or more phenomena”* (Hommel, 2019). When it comes to the field of neuroscience a theoretical framework used to motivate research should exemplify how (e.g., neural activity, or structures associated with a specific function) putatively generate a specific process. At present, considerations in cognitive neuroscience which neural substrates may contribute to a particular phenomenon. Yet, an idea of how this is done, what exactly neural structures do and how this is orchestrated is essential and needs closer consideration when deciding to examine neural data in close connection to an underlying cognitive theoretical framework (Beste, 2021).

### An example of efforts in the direction of reversed cognitive neuroscience research in psychiatry

Below, an approach is outlined reflecting a fruitful combination of expertise in neurology, psychiatry and neuroscience in the sense of a “synthetic approach” using a theory meeting the requirements of a mechanistic theory outlined above. This example is somewhat related to the example put at the beginning of the article (i.e., ADHD), because ADHD co-occurs with another neuropsychiatric condition—Gilles de la Tourette Syndrome.

GTS is a childhood-onset multifaceted neuropsychiatric disorder defined by several motor tics lasting more than 1 year (American Psychiatric Association, 2013). Tics range from simple movements/sounds like eye blinking or sniffing to complex movements/vocalizations, including utterances of words (Kleimaker et al., 2019). Coprophenomena, i.e., the execution of obscene gestures (copropraxia) or the utterance of swearwords (coprolalia), are also common. The first symptoms usually occur as motor tics around age 6 (Leckman, 2002). Most patients with GTS experience a pre-pubertal increase in tic severity, typically followed by a subsequent reduction or even complete remission toward the end of the second decade (Leckman et al., 1998). 59–85% of child/adolescent patients are tic-free or only have mild tics as adults (Pappert et al., 2003; Hassan and Cavanna, 2012). However, in the remaining 20%, symptoms continue or can become even more severe (Pappert et al., 2003; Müller-Vahl et al., 2010). The reason for this is currently unknown. Approximately 90% of all patients with GTS have co-morbidities, including attention deficit hyperactivity disorder (ADHD) (60%) (Freeman et al., 2000; Roessner et al., 2007; Robertson, 2011; Hirschtritt et al., 2015) and obsessive-compulsive disorder (41%) (Bloch et al., 2006). GTS has been perceived as a rare oddity predominantly manifesting as bizarre, socially unacceptable and inappropriate behavior in a few people of minimal medical relevance. This view has changed because (i) motor and phonic tics are very common in childhood (range of 3.4–24.4%) (Robertson, 2008), (ii) a considerable proportion fulfils diagnostic criteria for GTS, with an international lifetime prevalence of GTS between 0.5 to 1% (Robertson, 2008). (iii) most patients with GTS have “non-dramatic” symptoms that may not be noticeable at first sight. GTS has evolved from a peculiar fringe disease to a common and relevant neuropsychiatric disorder. However, an overarching framework of GTS is still missing, and the research on that disease has been dominated by a phenomenological approach with all the shortcomings outlined above. GTS is characterized by a repertoire of repetitive tics, which is relatively stable at a given time but *fluctuates* considerably over longer periods (Leckman, 2002; Robertson et al., 2017). There are tic undulations and migration over shorter (minutes, hours, days) and longer periods (weeks and months) throughout the disease (Jankovic, 1997). Thus, GTS phenomenology and severity can vary considerably inter- and intra-individually. Also, there are (near-) remissions and relapses with intervals of months or even years. Tic severity depends on *attention*. Focusing attention on tics significantly increases, and diverting attention to other tasks significantly decreases tic frequency (Brandt et al., 2015; Misirlisoy et al., 2015). Tics are partially *suppressible* and thus not completely involuntary. Tics may represent stimulus-driven sequential movements, are repetitive and patterned (Leckman and Riddle, 2000) and thus similar to “*habits*”. Patients with GTS have an increased habit formation tendency (Delorme et al., 2016), and tics may resemble over-learned behavior (Brandt et al., 2016). Patients have difficulty switching from ticcing to other actions until a tic is completed (Robertson, 2000; Leckman, 2002; Ganos et al., 2013). This resembles a central characteristic of processing capacity limitations during controlled, voluntary response selection (Luria and Meiran, 2005; Sigman and Dehaene, 2006) and places the tic-generating process close to processing constraints underlying “normal” response selection. *Sensory phenomena*, particularly urge preceding tics, represent a core feature of GTS (Leckman et al., 1993). Some patients with GTS consider their tics to react to their premonitory urges (Kwak et al., 2003; Schubert et al., 2021). Urge-tic correlations are quite variable (Schubert et al., 2021). There is a hypersensitivity to external stimuli (Cohen and Leckman, 1992), often leading to irritation and distraction (Cohen and Leckman, 1992; Belluscio et al., 2011), which is not due to altered sensory perception thresholds but seems to be caused by alterations of central processing as corroborated by experimental data (Orth et al., 2005; Nowak et al., 2005; Orth and Rothwell, 2009; Buse et al., 2016). These facets in GTS call for an explanatory concept encompassing action coding, perceptual processing and cognitive control.

This led us to propose conceptualizing GTS in a cognitive framework integrating perceptual, cognitive, and motor aspects of action, i.e., the Theory of Event Coding (TEC) (Hommel et al., 2001; Beste and Münchau, 2018). A well-established and validated mechanistic approach is taken to deconstruct a neuropsychiatric disorder. The Theory of Event Coding (TEC) (Hommel et al., 2001), represents a framework for perception-action coding proposing that perception and action are interconnected processes. Central is the idea of “binding”; i.e., how different features of a stimulus or features defining an action are integrated. TEC assumes that perceptions are specified/stored in so called “object files”, whereas specified/actions are stored in “action files”. Importantly, perceptions and actions are not stored and processed separately, but are related to each other in so-called “event files”. This leads to a binding between them (Hommel et al., 2001). Within an event file, stimulus and response features are bound to each other (Hommel, 1998a,b), so that event files could be conceptualized as a network of stimulus and response bindings (Hommel, 2009). We supposed that many facets of GTS could be explained by the event file concept and thus a theoretical approach rooted in basic cognitive science and psychology. Indeed, evidence suggests that this may be the case. Bindings between sensory and motor processes were found to predict tic frequency and thus a major clinical characteristic in diagnostic procedures (Kleimaker et al., 2020a,b; Weissbach et al., 2020; Mielke et al., 2021; Paulus et al., 2021) and it was shown how neurophysiological processes are changed in GTS ultimately leading to the view that GTS is actually not a classical movement disorders, but may rather reflect a disorder characterized by an altered integration of perception and action (Kleimaker et al., 2020a,b; Weissbach et al., 2020; Mielke et al., 2021; Paulus et al., 2021). Neurophysiological data were deliberately focused in these studies because the underlying event file concept likely reflects as a network of distributed representations (Hommel, 2009) likely constituted by one or a combination of the three following mechanisms: “Integration by Convergence”, “Integration by Correlation” or “Integration by Indexing” (Hommel, 2004). The first mechanism relies on neural units that are selective for the presence of particular sensory or motor feature combinations. The second mechanism relies on synchronizing the firing patterns of neural units representing features of the same event. Synchronicity could increase the impact of the synchronized unit on other processes (e.g., perceptual or response processes). The third mechanism may work by enhancing the firing rates of sensory related neural units. A common theme of these possible mechanisms leading to the emergence of an event-file is the relevance of oscillatory activity, and its synchronization, as captured by electrophysiology (Buzsáki et al., 2012). Therefore, the choice of neuroscience methods was directly constrained by the underlying (synthetic) cognitive theory. Of note, findings suggesting that also clinical aspects of this disorder (i.e., tics) can at least be partly explained by altered perception-action integration (Kleimaker et al., 2020b). Moreover, the role of habit formation in GTS, as a major facet leading to clinical symptoms in GTS, could also be accounted for (Takacs et al., 2021). In addition, the TEC-framing provided a means to explain why cognitive behavioral interventions in GTS are likely to be effective (Petruo et al., 2019, 2020)— mechanistic insights that could not be gained in all phenomenologically driven clinical trials previously. Thus, insights were gained through theory and not through clinical definition and phenomenology. This was made possible by translating theory-driven experiments into a clinical setting without assuming any kind of homogeneity in the phenomenology or the mechanistic causes generating it. This equals reversing the strategy of research in cognitive neuroscience in the mental health field. Importantly, when using the same theoretical background as used to examine and explain GTS to other neuropsychiatric disorders (e.g., ADHD), the approach taken directly reflects the advocated “synthetic approach” because the same principles are directly brought into a “different” diagnostic category and it is examined in how far this category reveals commonalities and differences to GTS. Especially considering the high-comorbidity between ADHD and GTS makes a synthetic approach starting from a cognitive theory useful. Importantly, clinical considerations and necessities can directly be incorporate in the novel (i.e., synthetic) research strategy. This is important because then clinical constraints to work with categorial diagnoses and innovative research can be linked with each other. The finding discussed above also show that a research strategy rooted in a “synthetic approach” in the sense that a theoretical framework rooted in cognitive science can be used to explain clinically relevant phenotypic variations in a disorder supposed to reflect dysfunctions of completely different processes (i.e., motor functions). Of note, this is also relevant from a societal perspective; i.e., how disorders are viewed by society. The taken approach helps to better conceptualize their heterogeneity and the relationship between conditions. The approach enables that individuals with “disorders” can be grouped and targeted with reference to alterations of basic processes rather than to observable symptoms that may or may not indicate a shared problem. Therefore, the approach allows a specification of a distribution of functioning of a process and determination of individuality on this continuum that will facilitate the replacement of categorizations (healthy vs. ill, disorder A vs. B etc.) with mechanism-based dimensional approach. To the extent that “atypical” or disorder-related processes can be explained through mechanisms that also determine “typical” (healthy) behavior, such an approach has societal relevance as it contributes to a de-stigmatization of (mental) atypicality.

### Political implications

The aspects discussed in this article have clear political consequences. Science-political aspects in cognitive neuroscience research and changing these will significantly impact the benefits of patients and society. There are at least two interrelated themes rooted in funding politics:

The first theme relates to whether phenomenological or synthetic approach research should receive increased funding. There are many good arguments for a more synthetic research strategy in cognitive neuroscience and mental health. However, more funding must be invested in the so-called synthetic approach. To the extent that such a research strategy is not implemented sustainably, the problems presented so far in Topics 1, 2, and 3 become even more manifest. However, it would be too short-sighted to limit a “re-thinking” of research strategy to whether a more phenomenological or mechanistic approach should be promoted. Instead, it is essential to reconsider the “necessity” in research to look only for differences between populations—including the currently neglected ones (cf. Topic 3). Commonalities between groups are at least as necessary as differences between groups. The former, in particular, must be given greater weight in funding agencies and scientific publishing. Funding should be expanded in the portfolio, and especially studies should be launched that aim to find similarities based on a solid theoretical framework rooted in basic cognitive neuroscience. Currently, such studies exist (but only in the direction of thinking) within the framework of “non-inferiority clinical trials”. However, they are non-existent in expanding the mechanistic understanding of mental health problems beyond phenomenology. The practice of “pre-registrations” (see problems associated with this in “Topic 2”) and registered reports can currently be seen more as getting “insurance” to publish potentially “negative results” anyway. This cannot be seen as a proactive approach to the scientific research process in a research branch in which the “standing” of the weak people in society can be strengthened in the long term by not finding differences. In this respect, there is a high responsibility on the part of research funding organizations to change the conditions for researchers and enable cognitive neuroscientists to get a more active role in efforts to help the weak people in society to get into a stronger position. Researchers must be given the opportunity, within the framework of their research strategy, to pursue much more actively questions of how far individuals with mental health problems are not different but similar to populations considered healthy. Adjustments in funding opportunities can sustainably shape how research is conducted in the field and it is (partly) a role of governmental agencies to direct research in a way that societal needs are met in indenture with scientific requirements moving a field forward. Actively promoting research efforts with specific funding instruments that follow a strategy reflected by the “synthetic approach” are needed. As outlined above, the difference it in importance between finding an effect or not (difference between groups of individuals suffering from mental health problems and individual without mental health problems) is moderated by the presence or absence of strong theoretical predictions. Promoting a synthetic approach based on strong theory-driven predictions by funding agencies will enable researchers to work on scientifically important questions for which any outcome of the work is conceptually meaningful. Importantly, through such means not only problems inherent to research will be addressed. Rather, societal aspects are also automatically taken into account, thus generating considerable added value from a change in research strategy.

Promoting a change in research strategy and questions related to funding policies in cognitive neuroscience also touch a social political level, namely that of so-called “patient empowerment”. Patient empowerment refers to enabling and strengthening people to set priorities and make decisions to maintain or regain their health, cope with an illness situation, and manage it on their own or with external help. Empowerment enables people to set priorities and make decisions to maintain or regain their health, cope with an illness situation, and manage it on their own or with external help. Cognitive neuroscientists can play a role in supporting patients suffering from mental health problems to empower themselves by providing information about their deficiencies and their abilities. At present, patient empowerment has several aspects, such as enabling “health literacy”, “motivation”, “participation and self-management”, as well as “patient-centeredness”. However, all of these aspects come with the flavor of deficiencies at the patient’s site and measures to overcome these deficiencies. It will be essential, and possible through the help of cognitive neuroscience research in the mental health field, to get patients in a more active position by shaping a different societal view into the direction of abilities associated with mental health conditions (Colzato et al., 2022a). Therefore, cognitive neuroscience can and should play a much stronger role in shaping the societal view on mental disorders. Yet, for this to become realistic cognitive neuroscientists must actively reshape their field of research and contribute to the understanding of mental health problems.

## Conclusion

One of the most important frontiers in the development of cognitive neuroscience in the field of mental health research is to reshape their research strategy and by this their role in psychiatric research. While the importance of cognitive neuroscience research in mental health is widely acknowledged, cognitive neuroscientists can play a more active role in shaping research. The central contribution and potential of cognitive neuroscientists is to strengthen theory-driven mechanistic research (using basic psychological concepts) with the likely benefit that robustness of research is increased and that the position of individuals with mental health problems in society is strengthened.

## Data availability statement

The original contributions presented in this study are included in this article/supplementary material, further inquiries can be directed to the corresponding author.

## Author contributions

CB conceptualized the work and wrote the manuscript.
